# Chemical composition, anti‐hypertensive properties, and sensory attributes of salt extracted from ash of *Hygrophilia schulli*

**DOI:** 10.1002/fsn3.4217

**Published:** 2024-08-29

**Authors:** Degsew Mengistu, Paulos Getachew

**Affiliations:** ^1^ Department of Biology Kotebe University of Education, College of Science and Mathematics Education Addis Ababa Ethiopia; ^2^ Center for Food Science and Nutrition Addis Ababa University Addis Ababa Ethiopia

**Keywords:** *common salt*, *Hygrophilia schulli*, hypertension, sodium–potassium ratio, vegetable salt

## Abstract

The traditional usage of salt taken from vegetables for the treatment of blood pressure and diabetes is seen in several regions of Ethiopia, particularly in Gambella. The aim of the study was to investigate the mineral content, anti‐hypertensive properties, and sensory attributes of salt obtained from the *Hygrophilia schulli*. The salt was extracted from the ashing of stems of *Hygrophilia schulli*. Optical Emission Spectroscopy with Inductively Coupled Plasma was used to identify macro‐minerals, micro‐minerals, and some toxic metals. Flame Atomic Absorption Spectroscopy was used to assess the levels of arsenic and mercury. The anti‐hypertensive property of the salt was determined in vivo using the Wistar rats. The extracted salt was rich in potassium and was deficient in sodium from the analyzed macro‐minerals. It was free from mercury, cadmium, chromium, and arsenic. Lead and nickel were below the Tolerable Weekly Intake Provisional specified by the European Union. Among carbonate and sulfate, chloride was the main anion in this salt. The salt was discovered to have a low taste characteristic compared to common salt. However, the taste attribute of the combination of vegetable and common salt in different proportions was better than common salt. Rats fed with salt from *Hygrophilia schulli* showed a statistically significant lower systolic, diastolic, and mean blood pressure compared to normal‐fed and common salt‐fed rat groups. Generally, this study suggests that *Hygrophilia schulli* salt has the potential to be a viable alternative to common salt, particularly for those suffering from hypertension and other related chronic conditions.

## Graphical abstract

The aim of the study was to investigate the mineral content and anti‐hypertensive properties. The extracted salt was rich in potassium, deficient in sodium from the analyzed macrominerals, and rich in iron and zinc from microminerals.

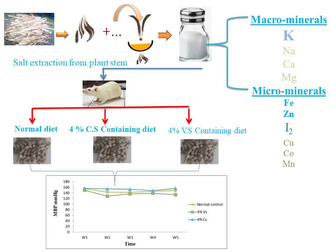

## 1 INTRODUCTION

Common salt (edible salt) that has been collected from the sea and rocks is an essential part of the diets of both domestic animals and humans. The two main ions found in common salt are sodium and chloride. Other substances, such as magnesium, calcium, iron, zinc, nickel, carbonate, and sulfate, are also present in common salts as impurities (Heydarieh et al., 2020a, 2020b). Since ancient times, salt has been used to improve taste and preserve and cure meat and fish. Because of these qualities, salts have significant role in human civilization (Williams, 2008).

Edible salt is produced from mined seas and rocks. The five top salt‐producing countries worldwide are China, the United States, Germany, and Australia. Salt production in Ethiopia has also a long history, and it is mainly practiced in the Afar region. Most of the salt is extracted from the Afar National Regional State (northeast Ethiopia) from Afdera Lake, near the border with Eritrea, which covers 95% of the overall production of salt. Ethiopia's estimated annual salt consumption is 350,000 tons, whereas Afdera alone has a 1.2 million tons production capability (Feyissa, 2011).

Worldwide and Ethiopian salt production is sufficient for prevailing demand. On the other hand, an excessive intake of common salt causes sodium ions in the blood to rise, which causes an excessive amount of water to enter the circulation. Blood pressure and the frequency of associated illnesses rise as a result of increased fluid flow in the blood vessels (MacGregor & de Wardener, 2002). Conversely, lower sodium salt consumption is important to use potassium‐rich salts extracted from vegetables as alternative sources to prevent chronic diseases. Accordingly, salt has been traditionally produced from wild and domestic vegetables by the lixiviation method; such salts are called vegetable or ash salt. Salts produced from ashing vegetables have been extracted and consumed by a large number of individuals in America, Central Africa, and Oceania. In Africa, about 158 plant species are utilized to make vegetable salt. For instance, in Central Africa, 35% of salt was produced from the ash of the plant parts (Mianpeurem et al., 2012). The salt is rich in potassium (i.e., potassium sulfate, potassium carbonate, and potassium chloride) and contains a lower amount of sodium which is good for preventing cardiovascular diseases and hypertension (Okayama et al., 2016).

Vegetable salt is also produced from *Hygrophilia schulli* and is consumed in the Gambella region of southwest Ethiopia. The plant is widely distributed throughout Amhara, Oromia, and other regional states of Ethiopia but the most common habitat for *H. schulli* is Gambella. *Hygrophilia schulli* is an unbranched herb with straight, curved thorns that belongs to the Acanthaceae family. In Gambella, Ethiopia, grows frequently in wetlands, ditches, and along the banks of the Baro River. The parts of the plant including salts are used as traditional medicine to treat different diseases by the local people (Tekulu et al., 2020).

*H. schulli* has many beneficial properties, such as anti‐oxidant, anti‐cancer, anti‐bacterial, anti‐fungal, hepatoprotective, and anti‐inflammatory properties (Nissanka et al., 2023). Different plant extracts play an important role in reducing stress and maintaining the regular homeostasis process (Sharma, 2022) and *H. schulli* is the major plant consumed by the locals in Ethiopia to extract salt traditionally. According to the local people, salts extracted from *H. schulli* are used to alleviate stomach pain, diabetes, and hypertension and to treat wounds. However, they lacked detailed information on when and how to begin consuming vegetable salts. As far as we are aware, no research has been done on ash salt production, consumption, and application of vegetable salt as an antihypertensive agent. Therefore, the study aimed to investigate mineral contents and toxic metals that are found in the ash salt from *H. schulli*, and evaluate the sensory quality of the salt as compared to common salt. This study also in vivo investigated the anti‐hypertensive properties of the ash salt using Wistar rats. The salt might aid in the creation of naturally occurring, low‐sodium salt substitutes that effectively lower the risk of hypertension and improve cardiovascular health.

## 2 MATERIALS AND METHODS

### 2.1 Sample collection and sampling site

The sample was collected from the Abobo District of Gambella, Ethiopia. The samples were packed in polyethylene (PE) plastic bags and transported to the laboratory of the Center for Food Science and Nutrition of Addis Ababa University, then it was oven‐dried (100°C) and burnt to obtain the ash. The common edible salt used in this study was randomly bought from open markets in Addis Ababa, Ethiopia. Regional State of the Gambella People's Nation is found in Ethiopia between latitudes 60 30′ and 80 30′ N and longitudes 330 00′ 350 45′ E, with total area of 26,000 Km^2^. Gambella is bound by Oromia Regional State to the North, Southern Peoples' Nations and Nationalities Regional State to the East, and the Republic of Sudan to the South and West (Awas et al., 2001).

### 2.2 Experimental area

Different food laboratories were used for the experiment. Kotebe Metropolitan University performed the salt extraction process and an analysis of the salt's anion content. Bless Agri Laboratory Service PLC analyzed the salt's toxic metals (Hg, Pb, Cd, Ni, and Cr), the Center for Food Science and Nutrition at Addis Ababa University examined the salt's taste attributes, the Ethiopian Leather Industry Institute analyzed major and trace metal content of the salt, and the Ethiopian Public Health Institute performed a salt antihypertensive test.

### 2.3 Salt extraction method

**FIGURE 1:**
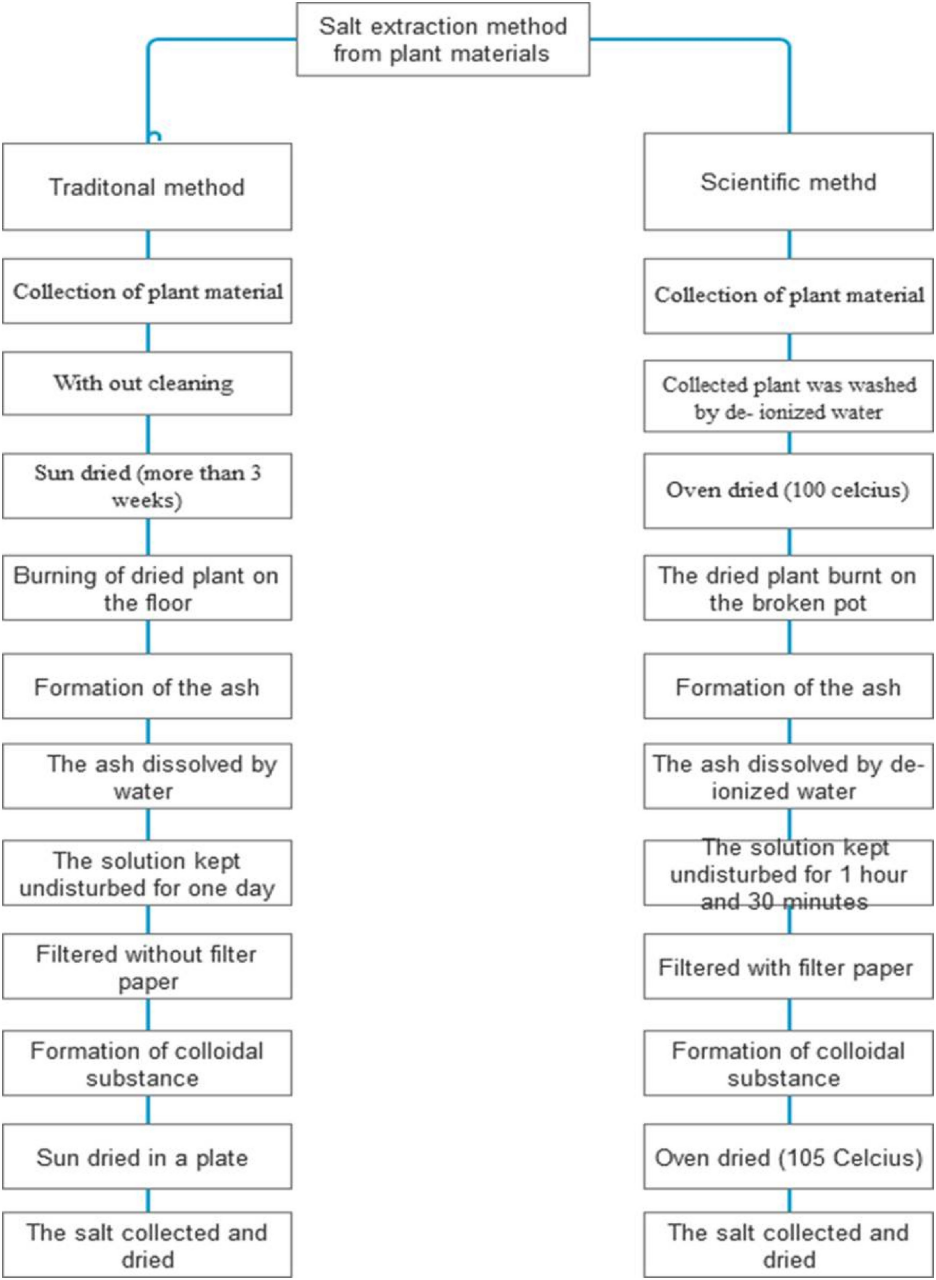
Traditional and scientific vegetable salt production methods (from the society).

The salt was extracted using the method of Mianpeurem et al. (2012), and the salt preparation method was adapted from the indigenous ash salt extraction method with some modifications (Figure 1). The ash was produced by burning the collected plant material (2935.47 g from the stem and 1320 g from the leaf). Six hours were needed to burn 2935.47 g of the plant stem to obtain the ash. However, the temperature, moisture content, burning surface area, and thickness/density of the stem can all be significant factors in totally ashing the plant material. Then, 100 g of ash from each plant sample was dissolved in 1.5 L of distilled water. The solution was thoroughly mixed and kept at room temperature for 1 h. Then, it was mixed again for 30 min to dissolve all salts in the ash. Afterward, the solution was passed through a filter paper (Watman International Ltd, 11.0 cm, Cat No. 10,936,211) to separate the salt and impurities. Finally, the filtrate was placed in a drying oven (Model 105, Industrial Estate, Ambala Cantt‐India) at 105°C until it crystallized; 500 mL of salt solution in 1 L beaker was crystalized in 20 h.

### 2.4 Analytical methods

#### 2.4.1 Quality factor determination of the salt

Quality factors including matter insoluble in water, moisture content, and pH of vegetable salt from *H. schulli* were determined. Water‐insoluble matter was determined using ES ISO 2479, moisture content was obtained using oven‐drying method (AOAC, 1998), and pH was measured using a 009 (1) pen‐type digital pH meter.

#### 2.4.2 Determination of anion composition

Anions (sulfate, alkalinity, and chloride) contents in the salt samples were determined. Sulfate as barium sulfate was determined by the gravimetric method, alkalinity as sodium carbonate was determined by AOAC (1998), and the chloride content of the salt was determined using the potentiometric titration method (EuSalt/AS 016–2005 test method).

#### 2.4.3 Macro‐mineral, micro‐mineral, and toxic metal analysis

The contents of selected macro‐minerals, micro‐minerals, and toxic metals (Pb, Cd, Ni, and Cr) were analyzed by Inductively Coupled Plasma‐Optical Emission Spectroscopy (Agilent 700 series, USA) using ISO11885:2012 test method. Arsenic was determined by graphite atomic absorption spectrophotometer (Model G8432A, Agilent Technology, Malaysia) and Hg by Cold Vapor Atomic Absorption Spectrophotometer (model G8436A, Agilent Technology, Malaysia) using ISO 17025: 2005 test method. Iodine was determined using the Association of Official Analytical Chemists (AOAC, 1998) guidelines. In this method, iodate was reduced to iodine (I_2_) and then titrated with sodium thiosulfate using a starch indicator.

#### 2.4.4 Sensory determination of the salt

The taste attribute of the salt was evaluated by paired comparison, multiple comparisons, and rating descriptive sensory test using a 7‐point hedonic scale (Franco et al., 2020). The sensory acceptability was done by 12 panelists for a paired comparison test, and 17 for multiple comparison and rating descriptive sensory tests. Untrained panelists (aged between 22 and 47) were randomly selected among staff and graduating class students of the Center for Food Science and Nutrition, Addis Ababa University, Ethiopia.

#### 2.4.5 Rice preparation for the sensory test

Rice preparation was done by using the method of Saavedra‐Garcia et al. (2015). For the paired comparison test, 100% common iodized salt and 100% vegetable salt were used. For multiple comparison tests, 20%, 40%, 60%, 80%, and 100% potassium‐enriched *H. schulli* salt and 100% sodium chloride as references were used in the rice preparation.

#### 2.4.6 Sensory test (evaluation)

Experimental samples were coded by three letters: the rice prepared with 100% vegetable salt was coded as BAB and the rice prepared with 100% sodium chloride was coded as ABB for paired comparison. BAA (20% vegetable salt), AAB (40% vegetable salt), ABB (60% vegetable salt), ABA (80% vegetable salt), and R (reference = 100% sodium chloride) for multiple comparisons sensory test. After orientation, two coded samples for paired comparison and five coded samples including reference for multiple comparison tests were given in random order to the tasters along with the cup of water to clean their mouths between sample tasting. The experiment then was done with rice using common salt and vegetable salt from *H. schulli* and the sensory evaluation test was done by selected panelists.

### 2.5 Anti‐hypertensive test

#### 2.5.1 Experimental animals and housing

The experiment was done using the method of Boegehold and Kotchen (1990). Four‐week‐old male Wistar rats (weighing between 200 g and 270 g) were purchased from the Ethiopian Public Health Institute (EPHI). The rats were housed at 22 ± 3°C room temperature with normal humidity (40%–70%). The feed was supplied to rats for 5 weeks after 2 weeks of adaptation period. Then, rats were randomly assigned to three groups: normal feed group (control) (group 1, n = 6), 4% *H. schulli* salt‐containing feed group (group 2, n = 6), and 4% common salt‐containing feed group (group 3, n = 6). Then, feed and tap water were provided ad libitum throughout 5 weeks of feeding time.

#### 2.5.2 Heating (warming)

The rats were picked up from the cage and held on hand for 30 s and then returned to the cage for 15 min. Afterward, each rat was removed from the cage and placed into the restraint tube, and transferred into heater (the part of tail‐cuff machine with the temperature maintained at 30 to 33°C). The restraint period lasted for 15 min. Once the rats within the restraint tube were heated, final blood pressure was measured by a tail‐cuff machine (Model 179, 23,924 Victory Blvd Woodland Hills CA91367, USA, blood pressure analyzer) (Wilde et al., 2017).

#### 2.5.3 Body weight and feed intake measurement

The body weight of the rat was measured once a week individually at the same time and group feed intake was estimated twice a week by measuring the feed remaining in the cages after 24 h. Then, feed efficiency ratio (FER) was calculated.

#### 2.5.4 Blood Pressure Measurement

After heating arterial blood pressure was measured by an indirect method using the tail‐cuff method. Ahead of the experiment, each rat was trained for 2 weeks until the blood pressure was steadily recorded with minimal stress in the restraint holder. Blood pressure reading (BPR) cuff tubing was secured in the notch on the top rear of the holder and finally, the cuff was attached to the controller (restrainer). The first measured baseline blood pressure was discarded and the mean of five subsequent measurements was recorded. Accordingly, systolic, diastolic, and mean blood pressures were measured weekly for 5 weeks by the indirect tail‐cuff method (Balaraman et al., 2006). Systolic and mean BP was taken from the reading (graph) of the machine and diastolic BP was calculated.

#### 2.5.5 Data analysis

The data were performed in triplicate and presented as means ± SE. Statistical comparisons of the mean values were performed by % for paired comparison and multiple comparison of sensory analysis, paired‐samples t‐test for rating sensory test, and analysis of variance (ANOVA), followed by Duncan's multiple‐range test using SPSS software (version 23) for quality factors, anion compositions, macrominerals, microminerals, toxic metal compositions, and antihypertensive test. Means were considered significantly different at *p* < .05.

#### 2.5.6 Ethical clearance

Ethical clearance for animal‐based experiments and sensory evaluation tests was approved by the College of Natural and Computational Science Institutional Review Board (CNS‐IRB) Committee.

## 3 RESULTS AND DISCUSSIONS

### 3.1 Percent of salt obtained from ash and raw materials of *H. schulli*

**FIGURE 2:**
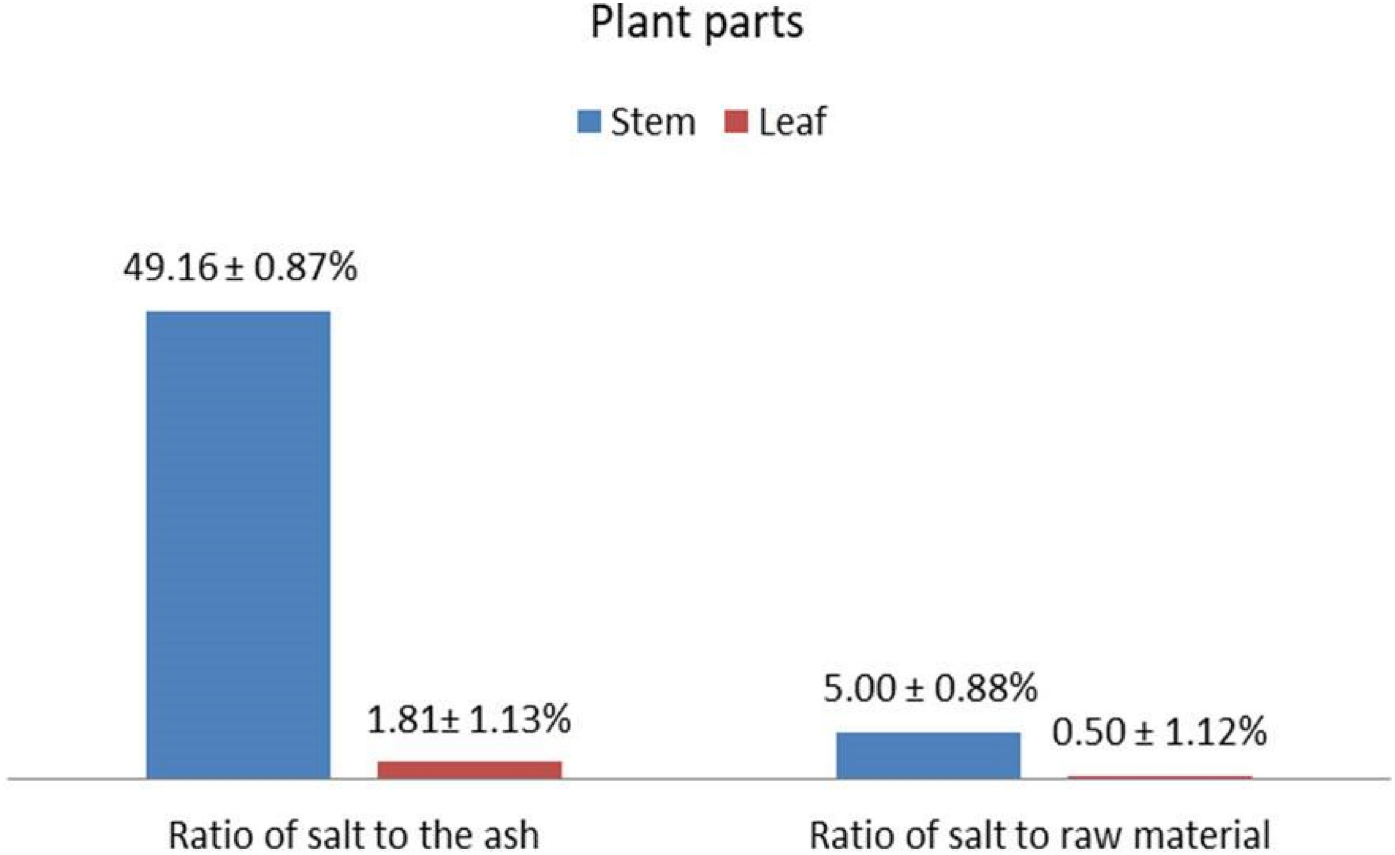
Percent of salt calculated from ash and raw materials of *H. schulli* (%).

The salt was extracted from the stem and leaf of the plant. The salt amount (%) was calculated from the total ash and raw material used for the preparation (Figure 2). A total of 2935.47 g of fresh *H. schulli* stem was measured. From this amount, 298.589 g of ash was obtained after kindling the pyre in open air. In the same way, from 1320 g of fresh *H. schulli* leaves, 363.8 g of ash was obtained. So, the average ash‐to‐raw materials were 10.2% and 27.6% from the stem and leaf of *H. schulli*, respectively. Similarly, the average ratio of salt to raw material from the stem and leaf of *H. schulli* was 5.0% (146.77 g) and 0.5% (6.6 g), respectively. The salt yield was higher when considering the ratio from the total ash (i.e., 49.16% and 1.81% from the stem and leaf of the plant, respectively) (Figure 2). According to my observation and the locals in Gambella, Ethiopia, the plant's stem is entirely ashed but the leaf is not, and the content of minerals like sodium and potassium in the stem of the plant is higher than in the leaf (Idris et al., 2011). Plant stems are a source of vegetable salt, as reported by Echeverri and Román‐Jitdutjaaño (2011) and Mianpeurem et al. (2012).

There are very few studies on vegetable salt extraction, composition, etc. to compare and contrast with this study. Among these few studies, a study by Chad reported the elemental composition of vegetable salt from four common plant species. Among the four species, 34.0% of salt from ash and 9.5% of salt from the raw stem of *H. auriculata* (Mianpeurem et al., 2012).

### 3.2 Quality factors of the salt

When assessing the quality of salt, it is crucial to take into account its moisture content, pH, and matter insoluble in water. The moisture contents of the vegetable and common edible salts were significantly different (*p* < .05), with common edible salt being more dried. The common salt's moisture content is less than what ES specifies (Table 1). In the meantime, the moisture level of the vegetable salt is within the acceptable range. Lack of anticaking agents during reprocessing, incomplete drying after washing, and processing season are all important factors that affect the moisture content of salt. For instance, a study in Morocco shows that the moisture content of salts was 1% during the dry season, 3–4% during the wet season, and 0.55% of moisture in common salt as reported by Zimmermann et al. (2004).

**TABLE 1:** Anion composition and quality parameters analyzed from vegetable salt.

Quality factors and anion composition	Salt samples		
	C.s	V.s	E.s/codex
Matter insoluble in water (%)	0.29 ± 0.004*	0.13 ± 0.01	1.00
Moisture content (%)	0.28 ± 0.01*	1.77 ± 0.05	0.50–4.00
pH	7.13 ± 0.12*	9.97 ± 0.03	7.00–8.00
Chloride ion (%)	59.80 ± 06*	12.43 ± 09	59.00–60.00
Sulfate (%)	0.10 ± .00*	0.67 ± .012	0.50
Carbonate (%)	0.28 ± 0.004*	3.82 ± 0.01	0.20

*Note*: Data are expressed as mean ± SEM (*n* = 3).

Abbreviations: C.s, Common salt; E.s, Ethiopian standard; V.s, Vegetable salt.

* Means in the same row are significantly different at *p* < .05.

Similar to moisture content, water‐insoluble matter in common edible salt can affect the stability of iodine. Contents of insoluble matter in water between the two salt samples were significantly different (*p* < .05). These amounts are within the accepted Ethiopian standard. The amount is higher in common salt than vegetable salt (Table 1). This might be due to the exposure of common salt during production to foreign materials like sand, dust, and ash. Since the production of vegetable ash is commonly in confined areas, there is a low chance of being contaminated with water‐insoluble matter.

The pH values of vegetables and common salt were significantly different (*p* < .05). The pH of the vegetable salt was higher than the common salt and the standard of Ethiopia (Table 1). This means that the pH of salt extracted from *H. schulli* is alkaline. Salts produced from the ash of plants are basic to strongly basic (pH ranging from 11 to 14). As a result, most people in the Witoto society use vegetable salts as an alkaline reagent to liberate alkaloids from plant diets (Echeverri & Román‐Jitdutjaaño, 2011). The alkaline pH of salt obtained from ashing plants may be due to high concentrations of potassium and/or sodium carbonate and hydroxides of potassium and sodium.

### 3.3 Anion composition of *H. schulli salt* and common salt

Carbonate, sulfate, and chloride ions were analyzed from the salt samples. In this study, there was a significant difference in carbonate content between the *H. schulli* salt and common edible salt (*p* < 0.05) (Table 1). Meanwhile, vegetable salt contained a higher amount of carbonate compared with both common salt and set standards. In commonly consumed *H. auriculata* salt in Chad, the content of carbonate ions was 213.5 ppm. Similarly, Echeverri and Román‐Jitdutjaaño (2011) reported carbonate amount in vegetable ash collected in Witoto Amazon ranged between 0.38% and 34.07% from different plant species. The value of this study lies in this range. The higher amounts of carbonate in the vegetable ash make the pH alkaline. As a result, in this study, the pH of the *H. schulli* salt was higher than that of the common salt (Table 1). High concentration of carbonate in the salt and sulfate ions in vegetable salt may be common salt is treated further to reduce the concentration of these anions and other minerals. The major components of common salt are sodium and chloride ions. Other minerals, sulfate, and carbonate ions in common salt are considered impurities (Heydarieh et al., 2020a, 2020b).

Sulfate contents in vegetable salt and common salt samples are also significantly different (*p* < 0.05). The concentration of sulfate ions of common salt and ash salt of *H. schulli* are 0.1% and 0. 67%, respectively (Table 1). Sulfate concentration of ash salt from *H. schulli* is higher than the Ethiopian standard specification limit (0.5%). Reports of different studies show that sulfate concentrations of ash salt obtained from vegetables are high. For example, Porters (1950) reported that in central Africa, salts from the ash of some vegetables have high concentrations of sulfate.

The major component of common edible salt is sodium chloride. From this major component, about 59% of the salt is chloride ion (Nafees et al., 2013). In this study, the chloride content of vegetables and common salt is significantly different at *p* < 0.05 (Table 1). However, the major components of salt from the ashing of vegetables are in the form of potassium chloride, potassium sulfate, and potassium carbonate. Hence, the chloride content of vegetable salt is much lower than that of common edible salts. However, in vegetable salt, chloride ion content is the highest compositions compared to carbonate and sulfate (12.43%).

There is a relationship between the concentration of sulfate, carbonate, and chloride ions and the perceived taste of the salts; with the “sweet” flavor of chloride being the preferred taste (Echeverri & Román‐Jitdutjaaño, 2011). Therefore, vegetable salt can be an alternative salt maintaining the expected taste qualities of salt.

#### 3.3.1 Macro‐mineral composition of the salt

Macro‐minerals analyzed in the salt were sodium, potassium, calcium, and magnesium. In this study, vegetable salt has lower sodium content than the common salt. According to Mianpeurem et al. (2012), a lesser amount of sodium ions is reported in a salt sample extracted from different plant species. The sodium amount reported in *Hygrophilia auriculata* was 6 mg/L; the value in this study was 7 g/L. In both studies, the sodium amount is lower than the potassium compared to the common salt. But, the sodium content found in this study is much higher than in the previous report. This variation may be due to soil type, sodium is released from many minerals that break down over time, from concentrated ruff of pesticides, fertilizers, and fossils of the soil.

For instance, there is variation in sodium and other mineral contents in vegetable salts based on the species and ecology. In a dietary approach to reduce hypertension (DARH), sodium‐to‐potassium ratio of less than 1 is recommended. A study reported that Na:K ratio had a significant effect on prevalence of hypertension. Therefore, people should be concerned about the Na:K ratio in their diet. According to this study, the amount of sodium is significantly (*p* < .05) lower in vegetable salt compared to common salt. For instance, the Na:K ratio for the vegetable and common salt was 0.05 and 164, respectively (Table 2). Na:K ratio of less than 1 has been recommended for optimal health, with higher ratios associated with elevated blood pressure and increased risk of cardiovascular disease. Therefore, the vegetable salt extracted from *H. schulli* was demonstrated to be a healthier alternative to the common salt. That means, increased potassium intake may protect against stroke, hypertension, and other conditions for which lower potassium intake has been associated (Zonoubi & Goli, 2021).

**TABLE 2:** Mineral composition and levels of toxic metals in common salt and vegetable salt derived from *H. Schulli*.

Minerals and toxic metals	Salt samples		
	C.s	V.s	E.s/codex
Macro‐minerals (%)			
Na	39.37 ± 0.09*	0.71 ± 0.01	N.s
K	0.24 ± 0.00*	13.40 ± 0.00	N.s
Ca	0.34 ± 0.00*	0.30 ± 0.00	0.5
Mg	0.15 ± 0.00	0.15 ± 0.00	0.5
Micro‐minerals (ppm)			
Co	0.08 ± 0.00	0.08 ± 0.00	N.s
Cu	<0.01	<0.01	2
Fe	34.81 ± 0.01	34.61 ± 0.00*	50
Mn	3.03 ± 0.03	1.70 ± 0.00*	N.s
Zn	33.30 ± 0.00	52.60 ± 0.00*	N.s
I_2_	ND	12.00 ± 0.56*	N.s
Toxic metals (ppm)			
Cd	<0.004	<0.004	0.50
Cr	<0.0075	<0.0075	2.00
Pb	0.61 ± 0.00*	2.27 ± 0.00	2.00
Hg	<0.03	<0.03	0.10
As	<0.20	<0.20	0.50
Ni	4.46 ± 0.00*	<0.015	N.s

*Note*: Data are expressed as mean ± SEM (*n* = 3).

Abbreviations: C.s, Common salt; E.s, Ethiopian standard; N.s, no standard; V.s, Vegetable salt.

* Means in the same row are significantly different at *p* < .05.

In the current study, potassium concentration in the vegetable salt was significantly higher than in the common salt (*p* < .05) (Table 2). However, the K concentration was much higher than the previous reports. It is obvious that the major component of common salt is sodium ion. About 10% of the dry weight of plant parts can be potassium ions. Enzyme activity, primary metabolism, stomatal function, growth, turgor regulation, and other important functions in the plant system depend on significant presence of potassium in the cytoplasm (Sustr et al., 2019). May be this explains why there was a higher potassium concentration in the vegetable salt compared to common salt. For instance, Mianpeurem et al. (2012) reported 47.5 mg/L K concentration in *H. auriculata* salt. Based on the the research by Weaver and Marr (2013), a key parameter is the relative ratio of Na:K, than the individual elements amount. Thus, a Na:K intake ratio of less than 1 has been recommended for optimal health. Therefore, a prolonged dietary imbalance with high DSI, where the Na:K is much higher than 1.2, would lead to an increased risk of hypertension and related CVD. In this study, the Na:K was 0.05, indicating that the salt produced from *H. schulli* has a significant role in reducing hypertension and other related diseases.

Calcium concentrations in the salt samples were significantly different at *p* < .05 (Table 2), but the difference was not as large as in the case of Na and K. According to the WHO standard, the level of calcium should not exceed the value of 5 g/kg in table salt. Both the common and vegetable salt in this study are within this limit. Also, a similar calcium concentration was reported from vegetable salt from Witoto, Amazon (0.39%). However, a much lower calcium concentration (20 mg/L) was reported by Mianpeurem et al. (2012). Both common and vegetable salts in this study also had a magnesium concentration that is within the set Ethiopian standard for edible salt (0.5%). Compared to the report by Mianpeurem et al. (2012), the magnesium content in the present study was higher, which is 0.15% for both common salt and salt of *H. schulli* (Table 2). Cardiovascular disorders also have been associated with an increased risk of magnesium deficiency (Lin et al., 2023).

### 3.4 Micro‐mineral contents of the salt

Among micro‐minerals, iron, zinc, iodine, cobalt, copper, and manganese were analyzed. As reported in Table 2, there is no significant difference in iron content between the common edible salt and vegetable salt extracted from *H. schulli* but concentration of zinc in salts extracted *H. schulli* is significantly higher compared to common salt (*p* < .05). Iron concentration in common salt is found between the ranges 12–273 mg/kg and zinc concentration was in between the ranges 4–9 ppm (Nafees et al., 2013). Iron concentration in vegetable salt produced from *H. auriculata* was 31 ppm (Mianpeurem et al., 2012). Therefore, ash salt extracted from *H. schulli* can be used as an alternative edible salt with reduced health risks being a good source of microelements like iron.

According to Mianpeurem et al. (2012), 0.007 ppm was the maximum concentration of zinc in salt sample extracted from *Zea mays*, which is a very low concentration as compared to the value (52.6 ppm) in this study. However, a high concentration of zinc (500 ppm) was reported from *Bactris humilis*, which is a known women's plant in Witoto. The salt is required for pregnant and lactating women (Merialdi et al., 1999) and is important in the production of insulin, which the fetus requires from the 4th month of gestation onwards (Echeverri & Román‐Jitdutjaaño, 2011). Similarly, in Gambella, Ethiopia, salt extracted from *H. schulli* is used as traditional medicine for women. Micro‐minerals like zinc and iron are also essential for human health and play a key role in increasing the bioavailability of other nutrients but people around the world suffer from malnutrition due to a shortage of these minerals (Berhanu Desalegn et al., 2022). Therefore, this vegetable salt produced from *H. schulli* can be a potential alternative to common salt being a good source of zinc besides the discussed health benefits and treating other zinc deficiency‐related diseases.

Iodine was determined from three sample groups (i.e., from noniodized common salt, iodized common salt, and vegetables salt from *H. schulli*). The concentration of iodine from none iodized common salt was not detected, iodized common salt was 69 ± 0.367, and vegetable salt of *H. schulli* salt was 12 ± 0.5552 ppm (Table 2). Similarly, 40 to 72 ppm of iodine was detected from iodized common salt (Vithanage et al., 2016). The source of iodine may come from the soil and the river. Iodine in rivers has two main sources: oceanic cyclic and iodine weathered from soils and rocks (Moran et al., 2002). In this study, 12 ppm of iodine was found in the salt extracted from *H. schulli*. Hence, besides being an alternative vehicle to USI, this salt can reduce the cost of iodization due to the inherent iodine content.

The other micro‐minerals quantified from the salt samples are cobalt, copper, and manganese. There is no significant difference in the cobalt concentration between the two salt samples (*p* > .05). Copper concentration was below the detection limit (BDL) of the ICP‐OES value of 0.01 mg/Kg. Similarly, copper concentration in the salt from *H. auriculata* was BDL of the instrument used in the report by Mianpeurem et al. (2012). In this study, the manganese concentration of the *H. schulli* and common salt were 3.03 and 1.70 ppm, respectively (*p* < .05) (Table 2).

### 3.5 Toxic metal compositions of the salt

Cadmium, chromium, lead, mercury, arsenic, and nickel were selected among toxic metals to identify from the salt samples. The concentration of cadmium (Cd) in the common edible salt and *H. schulli* salt was not detected, which was less than the value of 0.04 mg/Kg. International Agency for Research on Cancer has reported cadmium as a carcinogenic agent which is a major cause of kidney dysfunction. Cadmium is also a tremendously toxic metal that can cause lung cancer; produce bone defects and adverse effects like emphysema, bronchiolitis, and alveolitis in humans (Mwove et al., 2023). Thus, the food sanitary standard of Cd for edible salt concentration value of 2 mg/kg is set by the Ethiopian standard; the concentration in the present study is below the detection limit (Table 2).

Similarly chromium concentration in both the *H. schulli* and common salt was below the detection limit, which was less than the value of 0.0075 mg/kg (Table 2). Mianpeurem et al. (2012) reported a 0.007 ppm chromium concentration in salt extracted from *H. auriculata*. Hexavalent chromium is a hazardous metal that causes carcinogenic and mutagenic reactions in people (Goyer et al., 1950). According to the Ethiopian standard, the amount of chromium that is acceptable in regular edible salt is 2 ppm, which is higher than the one detected in this study (below the detection limit).

In this study, the concentration of lead in common and *H. schulli* salt was 0.61 and 2.27 ppm, respectively (Table 2) (*p* < .05). The maximum permitted level of lead in food‐grade salt is 2.0 ppm according to the Codex Legislation. The concentration in the *H. schulli* salt was higher than this value by 0.27 ppm. Due to this concern, Tolerable Weekly Provisional Intake (PTWI) was calculated to check the safety of *H. schulli* salt from lead contamination. The PTWI of lead is 25 mg/kg body weight by WHO. With the assumption that a 60 kg adult will on average consume 10 g salt per day, the weekly lead intake for one person through common edible and *H. schulli* salts is 0.0007 and 0.0026 mg/kg body weight, respectively. These values are 0.0028 and 0.0104% of the set PTWI value by WHO/FAO, respectively. According to PTWI, the lead content in the *H. Schulli* salt is, therefore, safe.

The concentration of mercury in common edible salt and ash salt from *H. schulli* was below the detection limit (<0.03 mg/kg) (Table 2). In a similar report by Mianpeurem et al. (2012), mercury concentration was below detection limit in the salt extracted from *H. auriculata*. The allowed mercury concentration in edible salt according to the codex and Ethiopian standard is 0.1 mg/kg. Both salt samples are below this limit. The concentration of arsenic in common edible salt and ash salt from *H. schulli* was also below the detection limit (<0.20 mg/kg). The salt consumed by human allowed arsenic amount according to ES is 0.5 mg/kg, in which both salts in the study were not detected.

As reported in Table 2, the concentration of nickel in vegetable salt of *H. schulli* was below the detection limit (<0.015 mg/kg) but the concentration of nickel in common salt was 4.46 mg/Kg. The permissible limit for nickel in edible salt is 0.16 mg/day. The PTWI of nickel is 35 mg/kg body weight (Nafees et al., 2013). With the assumption of 60 kg adult and consumption of 10 g salt per day, the weekly nickel intake for a person through common salt in this study is 0.0052 mg/Kg, which is less than PTWI set by WHO/FAO. Therefore, salt is safe to consume.

### 3.6 The sensory quality of salt extracted from *H. schulli*

#### 3.6.1 Paired comparison difference and preference tests

The result showed that rice cooked with 100% *H. schulli* salt had a distinguishable taste from 100% common salt‐cooked rice. All 12 panelists indicated taste differences between the two samples. Among the panelists, 83% preferred the rice prepared with 100% common salt and only 16.7% of the participants (only 2) preferred the rice prepared with 100% *H. schulli* salt. This might be due to the characteristics of the metallic and bitter taste of *H. schulli* salt as a result of its major components such as KCl. Besides, the participants were not vegetable salt consumers and thus were not familiar with the taste of vegetable salt. Therefore, for future successful use of vegetable salt (potassium‐rich salt), the addition of some effective taste‐improving agents (TIAs) has to be introduced to overcome its sensory drawbacks (Saavedra‐Garcia et al., 2015).

#### 3.6.2 Rating descriptive sensory evaluation of the salt

**FIGURE 3:**
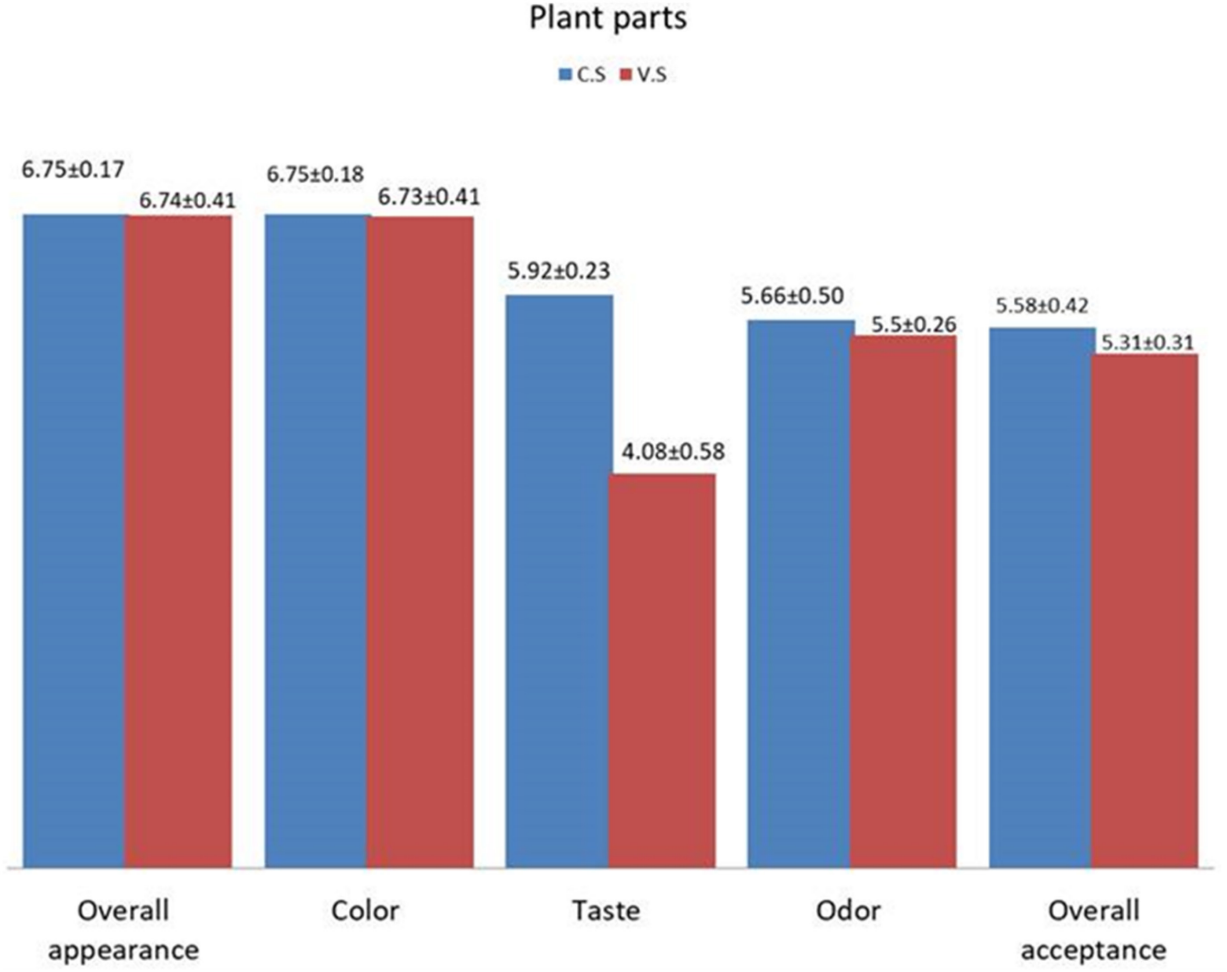
Results of a descriptive sensory test using common salt and *H. schulli* salt.

Descriptive sensory attributes of the two salts were evaluated by 17 participants. These sensory attributes were overall appearance, taste, color, odor, and overall acceptability. Taste is the essential sensory characteristic in edible salt and specifically is the major concern in vegetable salt due to its composition. The overall appearance, color, and odor of the two salt kinds in this investigation did not differ significantly (Figure 3). In contrast, there was a significant perceived taste difference between the two samples, in which the rice cooked with common salt had a better taste. There is a correlation between the concentration of chloride, carbonate, and sulfate ions with the perceived taste of the salts. For instance, salts that are rich in chloride and a low concentration of carbonate and sulfate are perceived as sweet and cool. Salts with a high carbonate content and low concentration of chloride and carbonate are perceived as strong and hot or biting. High concentrations of sulfate are perceived as sweet or insipid (Echeverri & Román‐Jitdutjaaño, 2011). The carbonate concentration of *H. schulli* salt in this study is high next to chloride ion. Maybe due to this reason, the taste perception was less accepted by the participants. However, it is possible to enhance the taste quality of the *H. schulli* salt through the addition of flavoring agents. Umami components, spices, vegetables, and other flavoring agents are some of the agents that can be utilized to enhance the taste of salt.

#### 3.6.3 Multiple‐comparison taste preference tests in different proportions of the two salts

For the multiple comparison tests, 20%, 40%, 60%, 80%, and 100% potassium‐enriched *H. schulli* salt and 100% sodium chloride (common salt) as reference were used in the preparation of rice. The result in Table 3 shows that the substitution rate of common salt with V.s increased, and the acceptability compared with R decreased (i.e., from 82% to 17%). Interestingly, the 40% V.s cooked rice had a 35% comparable taste acceptance with R cooked rice. Furthermore, the % of panelists detecting a difference as the V.s incorporation increased (i.e., from 17% to 82%). With 40 and 60% V.s replacement of common salt, 53 and 59% of the panelists perceived a better taste than R (100% common salt) cooked rice. These data indicated that even without additional taste improvers, by blending the V.s with C.s, an acceptable taste can be induced for the consumer and reduce intake of sodium ions.

**TABLE 3:** Multiple comparison taste preference tests in different proportions of the two salts.

Replacement level	Total participants	Comparable to R	Different from R	Better than R	Inferior to R
20% V.s	17	82%	17%	17%	0%
40% V.s	17	35%	65%	53%	12%
60% V.s	17	17%	82%	59%	23%
80% V.s	17	17%	82%	47%	35%

*Note*: Data are expressed as mean ± SEM (n = 3). C.s, Common salt; Vs, Vegetable salt from *H. schulli*, R, Reference (common salt) which was partially substituted with vegetable salt (20, 40, 60, and 80%). Means in the same row are significantly different at *p* < .05.

### 3.7 Antihypertensive test

#### 3.7.1 Effects of 4% salt extracted from *H. schulli* and 4% common salt on systolic, mean, and diastolic blood pressure of Wistar rats

Four‐week‐old male Wistar rats (weighing between 200 g and 270 g) were purchased from the Ethiopian Public Health Institute (EPHI). Rats were housed at 22 ± 3°C room temperature. The feed was supplied to rats for 5 weeks after 2 weeks of the adaptation period. The basic composition of the rat's diet is protein 14%, carbohydrate 48%, fat 4%, crude energy 16.5 KJ/g, and digestible energy 12.1 KJ/g (Oliva et al., 2017). 0.26% sodium chloride in a rat's diet is regarded as normal, 0.01% low salt, 4% medium‐high salt, and 8% high salt (Makhanova et al., 2008). Thus, meals containing 4% sodium chloride were selected for this investigation.

#### 3.7.2 Body weight and feed efficiency ratio (FER)

Rats fed with 4% V.s containing diet relatively gained more weight than the other groups. But the feed intake in this group was significantly the lowest compared with the other two feed groups (Table 4). This might be the less palatable nature of the V.s (metallic taste), which was observed in the sensory tests in this study. Similarly, the FER of 4% V.s diet‐fed mice was the lowest. Low FER means that the diet is more performed on the growth of rats.

**TABLE 4:** Body weight gain, feed intake, and feed efficiency ratio obtained from normal control, 4% common salt, and 4% vegetable salt for 5 weeks.

Fed group	*N*	Body weight gain (g)	Feed intake (g)	FER
Normal fed	6	80.33 ± 7.80	950.85 ± 2.50^b^	11.83 ± 1.21^b^
4% V.s	6	83.83 ± 2.18	922.07 ± 2.45^c^	11.07 ± 0.90^a^
4% C.s	6	67.83 ± 9.21	964.27 ± 1.05^a^	14.22 ± 0.60^c^

*Note*: Data are expressed as mean ± SE. Mean values in the same column with different superscripts are significantly different at *p* < .05.

Abbreviations: C.s, Common; FER, Fed efficiency ratio; V.s, Vegetable salt.

#### 3.7.3 Results of blood pressure

SBP and MBP were measured on the 1st week of the experiment before being fed with 4% common salt‐ and vegetable salt‐containing diets; this blood pressure was recorded as BP0. Accordingly, SBP0 ≤192 mmHg, MBP0 ≤150 mmHg, and DBP0 ≤ 132 mmHg were reported in the 1st week. Then, BP was measured every week for 5 weeks using a BP analyzer. The SBP and MBP were read directly from the pulse tracing, and DBP was calculated.

**FIGURE 4:**
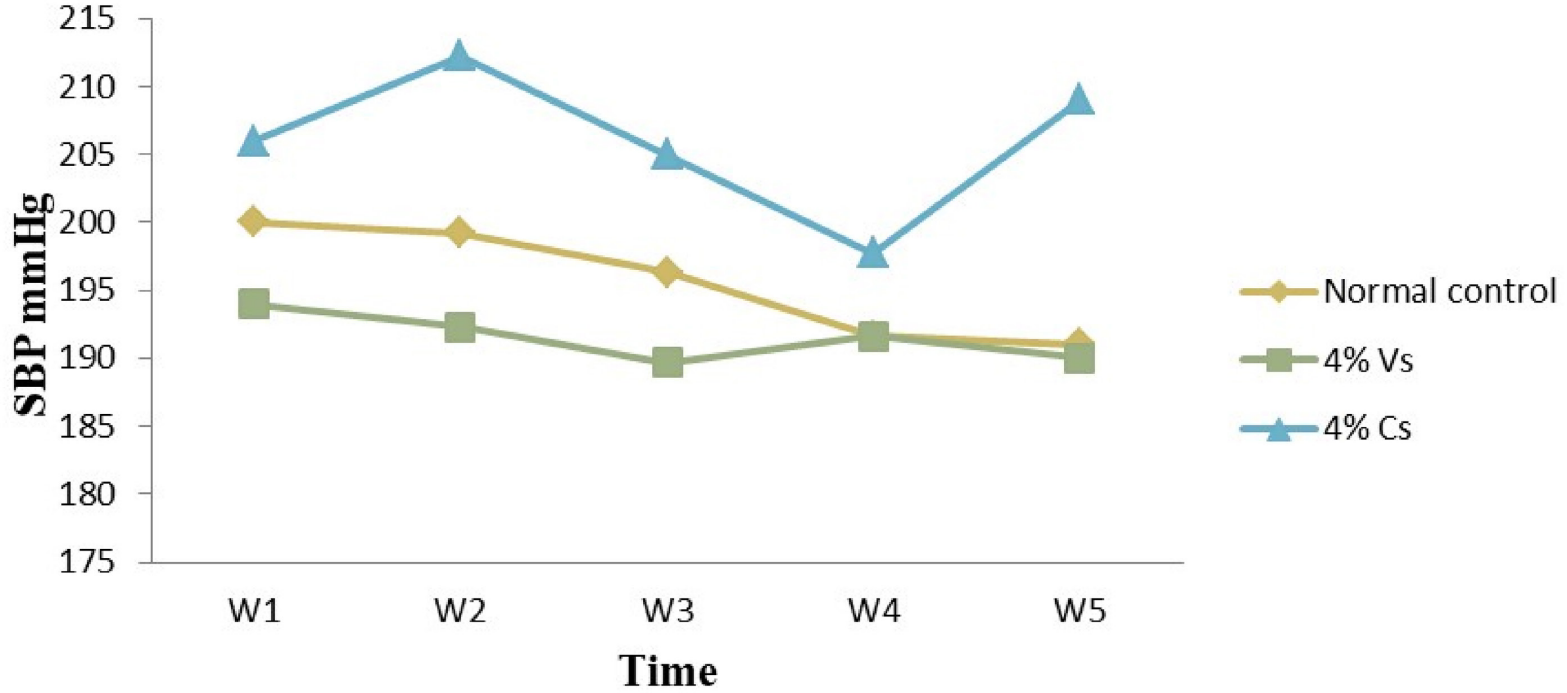
Weekly systolic blood pressure recorded from rats fed with 4% V.s, 4% C.s, and normal diets. V.s: Vegetable salt, C.s: Common salt, SBP: Systolic blood pressure.

**FIGURE 5:**
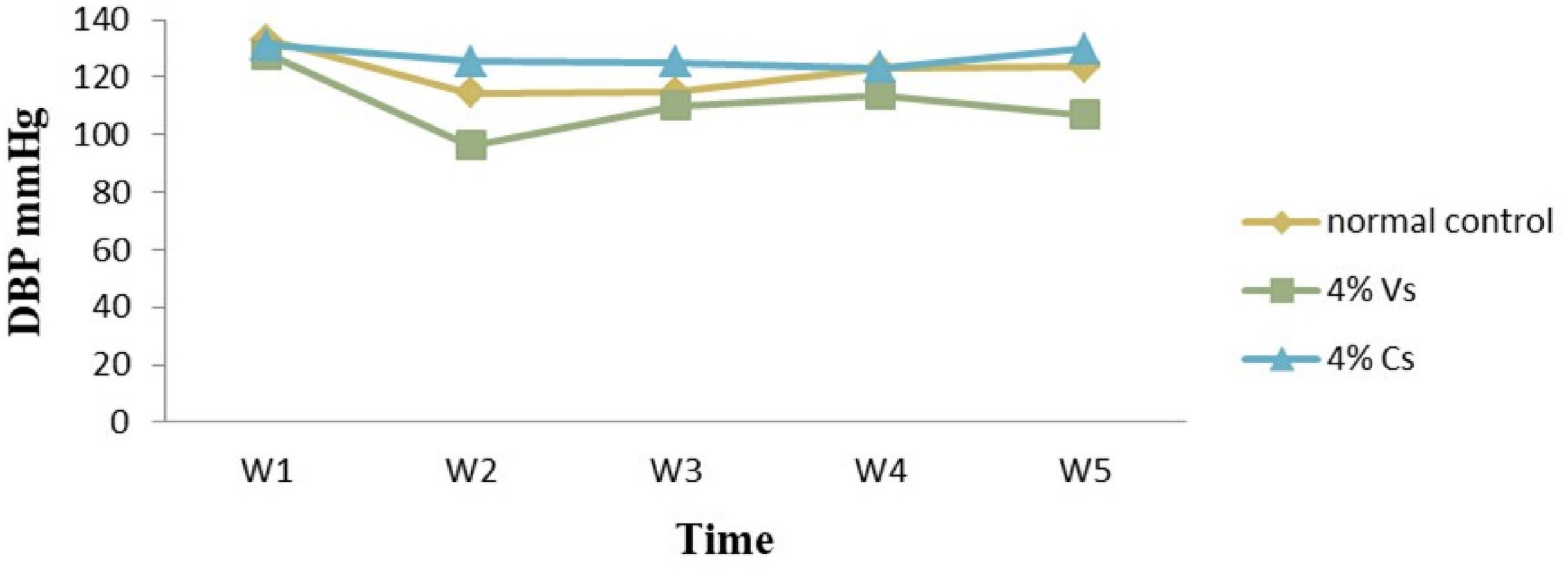
Weekly diastolic blood pressure recorded from rats fed with 4% V.s, 4% C.s, and normal diets, V.s: Vegetable salt, C.s: Common salt, DBP: Diastolic blood pressure.

As reported in Table 5, SBP, MBP, and DBP of the rats with 4% *H. schulli* salt‐containing feed decreased significantly (*p* < .05) as compared with the normal diet‐fed rats for 4 weeks. In contrast, in the 4% common salt‐incorporated diet‐fed rats, the SBP and DBP increased compared to the other groups (*p* < .05) (Figures 4 and 5). As discussed above, potassium‐to‐sodium ratio was higher in the *H. schulli* salt compared to the common salt in this study. Hence, the reduced sodium and increased potassium intake might contribute to significant systolic and diastolic blood pressure reduction (Baldo et al., 2014).

**TABLE 5:** Average Systolic, mean, and diastolic blood pressure of rats fed a diet containing 4% salt extracted from *H. schulli* and 4% common salt and normal diet.

Group	*N*	SBP (mmHg)	MBP (mmHg)	DBP (mmHg)
Normal control	6	195.6 ± 0.88^b^	146.4 ± 0.50^b^	121.5 ± 0.66^b^
4% V.s	6	189.5 ± 1.12^c^	137.7 ± 1.87^c^	111.3 ± 1.40^c^
4% C.s	6	205.8 ± 0.99^a^	153.5 ± 1.07^a^	127.0 ± 1.32^a^

*Note*: Data are expressed as mean ± SEM (n = 6), mean values in the same column with different superscripts are significantly different at *p* < .05.

Abbreviations: C.s, Common salt; MBP, Mean blood pressure; SBP, Systolic blood pressure; V.s, Vegetable salt.

On the weekly basis of BP measurement, the SBP was always in the order of 4% C.s > normal diet >4% V.s‐fed rats between weeks 1 and 5. This is again related to the high potassium‐to‐sodium ratio in the *H. schulli* salt compared to common salt. In all the diet groups, the SBP of the rats decreased until week 4, then again the BP raised in the 5th week.

## 4 CONCLUSION AND RECOMMENDATIONS

### 4.1 Conclusion

The major composition of the salt extracted from *H. schulli* was potassium from macro‐minerals and chloride from anions, and the sodium–potassium ratio of the *H. schulli* salt was less than 1. The salt was also found with non detectable concentrations of toxic metals like Cd, Cr, As, Ni, and Hg and the detected concentrations of Pb were very low as compared with the provisional tolerable weekly intake of the metals. The anti‐hypertensive property of the salt was further proved through in vivo Wistar rat feeding experiment. The rats fed with the 4% *H. schulli* diet had significantly lower systolic and diastolic blood pressure compared to normal and common salt‐containing diet‐fed rats; this is because of the high content of potassium. The sensory acceptability of *H. schulli* salt increased when it was blended with different amounts of common salt.

### 4.2 Recommendations

Anti‐hypertensive properties should be investigated further and other health benefits like anti‐diabetic and anti‐microbial activities should be studied in detail. Studies to improve the taste of the salt more than the acceptability rate in the present value, the shelf stability of the salt, and the potential of this salt as a vehicle of universal salt iodization should be carried.

## AUTHOR CONTRIBUTIONS

**Degsew Mengistu:** Conceptualization (equal); data curation (lead); formal analysis (lead); funding acquisition (lead); investigation (lead); methodology (lead); resources (lead); software (lead); validation (equal); visualization (equal); writing – original draft (lead); writing – review and editing (lead). **Paulos Getachew:** Conceptualization (equal); data curation (supporting); formal analysis (supporting); investigation (supporting); methodology (supporting); software (supporting); supervision (lead); validation (equal); visualization (equal); writing – original draft (supporting); writing – review and editing (supporting).

## Data Availability

All data and resources regarding this manuscript are available from the corresponding authors.
